# Supplementary material for: Molecular Clustering of Metabolic Dysfunction-Associated Steatotic Liver Disease Based on Transcriptome Analysis
Source: Diagnostics (Basel). 2025 Jan 31;15(3):342. doi: 10.3390/diagnostics15030342 (PMC11817575; doi:10.3390/diagnostics15030342)
Supplement: Supplementary file 1 [file diagnostics-15-00342-s001.zip › diagnostics-3388422-supplementary.pdf]

Supplementary Table S1. Baseline characteristics of MASLD.

	Healthy (n=62)	MASLD without MASH (n=76)	MASLD with MASH (n=26)	*P* value
Age (years)	45.5±11.3	46.3±13.8	43.6±12.1	0.643
Male sex, n (%)	35.5	43.4	34.6	0.561
Hypertension (%)	4.8	30.3	30.8	<0.001
Type 2 diabetes (%)	6.5	18.4	46.2	<0.001
Body weight (kg)	65.0±14.0	87.4±35.6	110.3±42.1	<0.001
BMI (kg/m^2)	23.6±3.9	28.9±6.8	33.3±7.3	<0.001
Waist circumference (cm)	82.4±10.3	95.0±17.2	106.5±17.7	<0.001
SBP (mmHg)	119.8±13.7	128.6±16.1	131.2±14.6	0.001
AST (IU/L)	20.6±9.2	25.9±14.6	32.8±17.8	0.001
ALT (IU/L)	19.3±13.4	30.4±22.4	38.0±25.3	<0.001
GGT (U/L)	34.0±41.5	53.9±81.3	54.0±54.0	0.164
Triglyceride (mg/dL)	114.3±62.4	177.5±126.6	183.4±90.4	0.001
HDL-cholesterol (mg/dL)	58.6±16.0	52.2±14.4	43.9±12.4	<0.001
Fasting glucose (mg/dL)	98.8±17.1	104.7±19.8	117.2±34.2	0.002
HbA1c (%)	5.5±0.6	5.9±0.9	6.4±1.3	<0.001
CAP(db/m)	226.8±58.0	270.7±61.0	324.6±25.0	<0.001
VCTE (kPa)	4.3±1.4	4.8±1.8	9.2±6.0	<0.001
Steatosis	0.0±0.0	1.1±0.5	2.2±0.7	<0.001
Inflammation	0.0±0.0	0.7±0.6	1.7±0.5	<0.001
Ballooning	0.0±0.0	0.5±0.6	1.6±0.5	<0.001
NAFLD activity score	0.0±0.0	2.3±1.0	5.5±0.7	<0.001
Fibrosis	0.6±0.5	1.3±0.6	1.9±0.5	<0.001

Data are mean ± SD or n (%). BMI; body mass index, SBP; systolic blood pressure, AST; aspartate aminotransferase, ALT; alanine aminotransferase, GGT; glutamyl transferase, HDL; high density lipoprotein, CAP; controlled attenuated parameters, VCTE; vibrated controlled transient elastography, NAFLD; non-alcoholic fatty liver disease. MASLD; metabolic dysfunction associated steatosis liver disease. MASH; metabolic dysfunction associated steatohepatitis. P value < 0.05 by ANOVA and chi-square test.

Supplementary Table S2. Clinical parameters according to molecular phenotype.

	Healthy	MASLD			*P* value
		Cluster I	Cluster II	Cluster III	
Age (years)	48.3±11.3	43.3±9.5	47.2±13.3	43.3±12.7	0.189
Male sex, n (%)	33.3	27.3	53.8	64.3	
Hypertension	6.7	0.0	30.8	57.1	
Type 2 diabetes	6.7	0.0	23.1	50.0	
Body weight (kg)	62.7±15.5[a]	66.9±24.1[a]	81.8±20.7[b]	115.8±44.0[c]	<0.001
BMI (kg/m^2)	22.7±3.4[a]	23.5±3.5[a]	28.9±4.8[b]	34.0±8.2[c]	<0.001
Waist circumference (cm)	80.1±9.8[a]	81.0±8.7[a]	95.2±13.9[b]	108.2±18.5[c]	<0.001
SBP (mmHg)	117.5±11.6[a]	119.5±11.8[a]	130.4±17.7[b]	130.6±15.7[b]	<0.001
AST (IU/L)	22.3±11.1	22.7±16.4	26.6±13.9	27.6±14.9	0.290
ALT (IU/L)	20.0±16.1	25.0±23.1	32.1±22.3	31.1±21.0	0.067
GGT (U/L)	35.4±39.3	44.8±84.2	52.4±63.2	39.0±36.1	0.620
Triglyceride (mg/dL)	106.5±54.1[a]	127.0±71.4[a]	157.6±86.9[a]	206.1±151.7[b]	<0.001
HDL-cholesterol (mg/dL)	59.9±16.5[a]	56.7±15.5[a]	50.8±14.8[b]	45.3±10.0[c]	<0.001
Fasting glucose (mg/dL)	101.9±20.5[a]	96.6±12.5[a]	107.7±28.1[a]	112.0±24.0[b]	0.029
HbA1c (%)	5.6±0.6[a]	5.3±0.5[a]	5.9±0.7[b]	6.3±1.2[c]	<0.001
CAP(db/m)	229.5±54.5[a]	240.7±48.3[a]	276.9±73.1[b]	295.5±46.5[b]	0.002
VCTE (kPa)	4.5±1.4[a]	3.8±1.1[a]	5.4±2.1[b]	6.8±4.8[c]	0.001
Steatosis	0.1±0.4[a]	0.5±0.6[b]	1.3±0.9[c]	1.3±0.7[c]	<0.001
Inflammation	0.0±0.0[a]	0.3±0.5[b]	0.6±0.7[b]	1.4±0.6[c]	<0.001
Ballooning	0.0±0.0[a]	0.2±0.5[a]	0.4±0.6[b]	1.1±0.7[c]	<0.001
NAFLD activity score	0.1±0.4[a]	1.1±1.4[b]	2.3±2.0[c]	3.8±1.7[d]	<0.001
Fibrosis	0.5±0.5[a]	0.8±0.5[b]	1.2±0.6[c]	1.8±0.4[d]	<0.001

Data are mean ± SD or n (%). BMI, body mass index; SBP, systolic blood pressure; AST, aspartate aminotransferase; ALT, alanine aminotransferase; GGT, glutamyl transferase; HDL, high-density lipoprotein; CAP, controlled attenuated parameters; VCTE, vibration-controlled transient elastography; NAFLD, nonalcoholic fatty liver disease. Metabolic dysfunction is associated with steatotic liver disease. MASH: metabolic dysfunction associated with steatohepatitis. P-value < 0.05, by ANOVA and chi-square test.
